# Fecal level of butyric acid, a microbiome-derived metabolite, is increased in patients with severe carotid atherosclerosis

**DOI:** 10.1038/s41598-022-26759-x

**Published:** 2022-12-26

**Authors:** Kristine Stø, Jørgen Valeur, Thor Ueland, Gunn Helen Malmstrøm, Vigdis Bjerkeli, Marius Trøseid, Johannes R. Hov, Kristian Holm, Beate Vestad, Bente Halvorsen, Mona Skjelland, Karolina R. Skagen

**Affiliations:** 1 Institute of Clinical Medicine, University of Oslo, Postboks 1171, 0318 Oslo, Norway; 2 Department of Neurology, Oslo University Hospital, Oslo, Norway; 3 Unger-Vetlesen Institute, Lovisenberg Diaconal Hospital, Oslo, Norway; 4 Research Institute of Internal Medicine, Oslo University Hospital, Oslo, Norway; 5 Section of Clinical Immunology and Infectious Diseases, Oslo University Hospital, Oslo, Norway; 6 Department of Transplantation Medicine, Norwegian PSC Research Center, Oslo University Hospital, Oslo, Norway; 7 Section of Gastroenterology, Department of Transplantation Medicine, Oslo University Hospital, Oslo, Norway

**Keywords:** Microbiology, Neuroscience, Biomarkers, Neurology

## Abstract

The short-chain fatty acid (SCFA) butyric acid maintains a healthy gut barrier and vascular endothelium. We aimed to investigate the association between fecal butyric acid, carotid atherosclerosis and risk factors for ischemic stroke. Patients with severe carotid atherosclerosis (i.e. ≥ 50% stenosis) (n = 43) were compared with healthy controls (n = 38). We analyzed fecal SCFAs by gas chromatography, microbiota composition by 16S rRNA sequencing, markers of gut barrier damage and inflammasome activation by immunoassay, and plasma SCFAs by ultra-high performance liquid chromatography-tandem mass spectroscopy. Patients had higher fecal butyric acid level (*p* = 0.024), along with increased functional potential of microbial butyric acid production (*p* = 0.031), compared with controls. Dietary fiber intake was comparable. Patients had higher levels of gut barrier damage markers CCL25 and IFABP, and the inflammasome activation marker IL-18, whereas plasma level of butyric was similar. Increased fecal butyric acid was associated with higher BMI, waist-hip ratio, HbA1c, CRP and leukocyte count. Contrary to our hypothesis, patients with severe carotid atherosclerosis had higher fecal butyric acid level, and increased microbial production, compared with controls. Gut barrier damage in patients might indicate decreased absorption of butyric acid and hence contribute to the higher fecal level.

## Introduction

Accumulating evidence suggests that dysbiosis—an imbalance of the microorganisms in the gut—plays a role in cardiovascular disease (CVD), including atherosclerosis^1^. Decreased abundance of microbes with capacity for producing butyric acid have been identified as a characteristic of gut microbiota in patients with atherosclerosis^2–4^, hypertension^5^, and heart failure^6^. The short-chain fatty acid (SCFA) butyric acid is a gut microbiome-derived fermentation product from dietary fibers and resistant starch with anti-inflammatory properties^1^, and is suggested as a possible link between the gut microbiota and atherosclerosis. Diets rich in fibers and starch reduce type 2 diabetes mellitus, obesity, ischemic stroke and CVD, and increased butyric acid and other SCFAs are considered key mediators for this benefit^7,8^. Butyric acid regulates several steps in the progression of atherosclerosis such as the antioxidant effect of NF-kB in endothelial cells, and macrophage-mediated lipid metabolism^9^. Furthermore, butyric acid may slow the rate of carotid intima thickening (IMT) by inhibiting the Nod-like receptor pyrin domain 3 (NLRP3) inflammasome in endothelial cells thereby reducing rate of progression from increased IMT to atherosclerosis^10^.

Fecal SCFA concentrations will depend on dietary intake of fibers and starch (i.e. substrates for fermentation), microbial fermentation capacity (production) and host factors (e.g. transit time and intestinal absorption). Butyric acid producing bacteria display high abundance in adults^11^, and most are members of the *Lachnospiraceae* and *Ruminococcaeae* families. The dominating production pathway is the carbohydrate-fueled pathway (pyruvate fermentation to butyric acid) where *Faecalibacterium prausnitzii* is a major contributor^12^.

SCFAs are rapidly absorbed in the colon and less than 5% is excreted in feces^13,14^. Increased intestinal permeability is observed in CVD, and is associated with enhanced systemic inflammation, potentially via leakage of bacterial components into the circulation and altered immune interactions in the gut^15^. It is proposed that chronically reduced levels of butyric acid contribute to gut barrier disruption^16^ and that SCFA treatment can reverse these defects^17^. Evidence suggests that NLRP3 inflammasome activation plays a role in the pathogenesis of impaired gut barrier as well as atherosclerosis^18^. Both loss of integrity in the epithelial barrier and bacterial translocation may enhance inflammatory processes in the intestinal mucosa where different lymphocyte subsets are important regulators of immune responses^19^. Multiple inflammatory markers are dysregulated in carotid atherosclerosis. However, in our study we chose markers that are specifically related to gut inflammation.

Based on the known association of SCFAs to inflammation and atherosclerosis, and the proposed interplay with gut barrier dysfunction, the aim of this study was to investigate if fecal level of butyric acid could be related to presence of carotid atherosclerosis and established risk factors of ischemic stroke. To the best of our knowledge, this is the first to assess actual levels of fecal SCFAs in patients with severe carotid atherosclerosis. The relatively unexplored field of fecal SCFAs is of great clinical interest when considering the gut as a possible future target for interventions to prevent symptomatic CVD. We hypothesized that patients with atherosclerosis would have lower level of fecal butyric acid and less butyric acid producing bacteria, evidence of gut dysbiosis and increased inflammation. This hypothesis was evaluated in a cohort of patients with severe carotid atherosclerosis and healthy controls. Fecal and plasma levels of SCFAs were measured, microbiota composition and function assessed, and markers of gut barrier damage and inflammasome activation evaluated.

## Results

### Baseline characteristics

The 43 patients with severe carotid atherosclerosis (i.e. ≥ 50% stenosis) and 38 healthy control subjects who provided fecal samples, were included (Table 1). All participants’ fecal samples were available for SCFA analysis, 78 were available for analysis for plasma markers of gut barrier damage and inflammasome activation, 64 for 16S rRNA analysis in feces, and 48 for SCFA in plasma (by Metabolon). Patients were older and had more pronounced cardiometabolic profile as reflected by their hypertension, dyslipidemia, diabetes, and increased waist-hip ratio, c-reactive protein (CRP) and leukocyte counts. Participants reported no significant differences in dietary intake of fibers or starch, or in Bristol stool scale (reflecting transit time) between the groups. Patients more frequently used antibiotics, antiplatelets and statins. Baseline characteristics in the subgroup populations matched the original population numerically for all variables, with some variation in p-values due to reduced power (Supplemental Table 1). When comparing levels of butyric acid and relevant butyric acid producers at genus level (more details in “Gut microbiota composition and function”) between sexes and users and nonusers of statins, antibiotics and platelet inhibitors, as well as correlating for age (analysis restricted to patients so the groups were comparable), the only significant difference we observed was for *Lachnospiraceae UCG-003* in users of antibiotics (Supplemental Table 2).

**Table 1:** Baseline characteristics of patients and controls.

	Patients N = 43	Controls N = 38	*p*-value
Age, (years)*	72.5 (6.3)	67.2 (7.9)	0.001
Male sex	44.2 (19)	28.9 (11)	0.156
Body mass index (kg/m^2^)	25.5 (3.8)	24.8 (3.6)	0.360
Waist-hip ratio (cm/cm)	0.96 (0.08)	0.89 (0.07)	0.001
Hypertension	79.1 (34)	21.1 (8)	< 0.001
Type 2 diabetes mellitus	25.6 (11)	0 (0)	0.001
Hypercholesterolemia	55.8 (24)	18.4 (7)	0.001
Anti-platelet treatment	81.4 (35)	10.5 (4)	< 0.001
Statin treatment	79.1(34)	10.5 (4)	< 0.001
C-reactive protein, (mg/L)*	3.4 (5.2)	1.2 (0.98)	0.014
Leukocyte count, (10^9^/L)*	7.8 (1.9)	5.1 (1.1)	< 0.001
Total cholesterol, (mM)*	4.1 (1.0)	5.2 (0.88)	< 0.001
LDL cholesterol, (mM)*	2.2 (0.85)	3.1 (0.85)	< 0.001
HbA1c (%)*	5.8 (0.88)	5.3 (0.28)	0.001
Antibiotics last 3 months	26.5 (9)	0 (0)	0.003

Values are given as % (n) or *mean (SD).

### Fecal and plasma SCFAs in patients compared with controls

**Figure 1:**
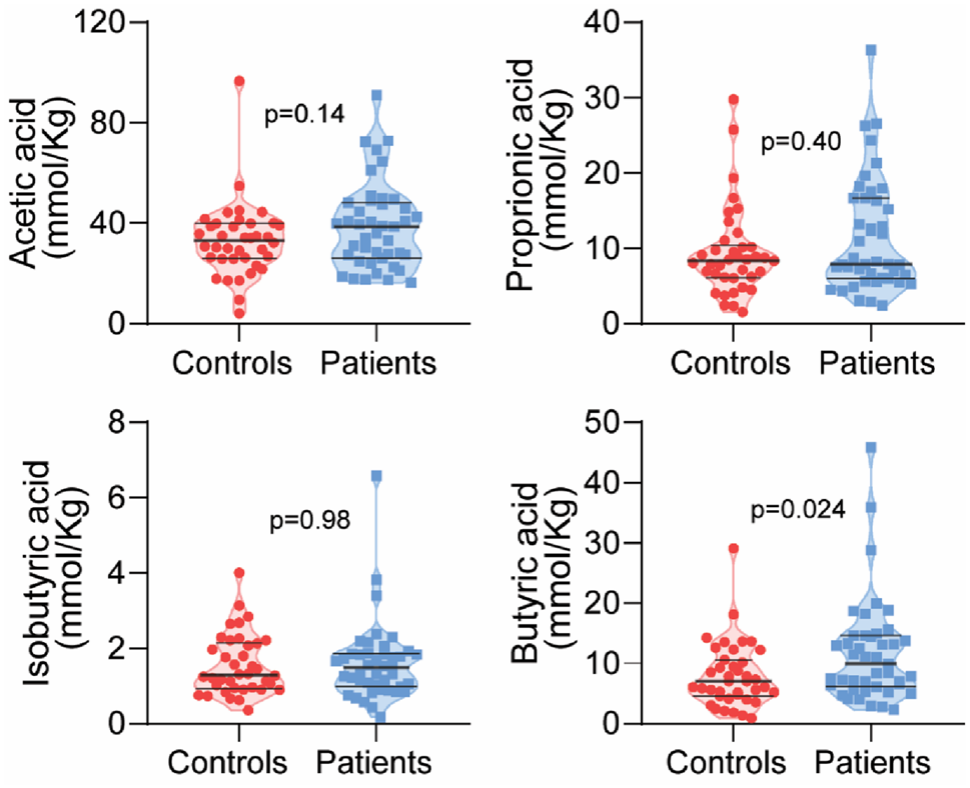
Violin plots showing levels of the SCFAs acetic, propionic, isobutyric and butyric acids in fecal samples in patients and controls.

In fecal samples, we found significantly higher level of butyric acid in the patient group (10.0 mmol/kg (2.2–45.8) vs. 7.0 mmol/kg (0.9–29.11); *p* = 0.024), while acetic and propionic (and isobutyric) acids had similar concentrations (Fig. 1). In plasma, butyric and isobutyric acids were analyzed as a total (n = 48, see “Methods”), and there was no significant difference between the groups (*p* = 0.747).

### Gut microbiota composition and function

For assessment of gut microbiota composition, fecal samples from 32 patients and 32 controls were available for analysis. There were no differences in the global microbiota composition as measured by beta diversity (unweighted unifrac) or in the intra-individual (alpha) diversity between the groups (observed features and Faith's phylogenetic diversity, data not shown).

After filtering out bacterial genera present in less than 20% of the samples, 141 genera were included in the analysis. The top 10 bacterial genera (average abundance) in patients and controls did show not major differences (Supplemental Fig. 1). In total, 13 genera were different between the two groups at p < 0.05 (Supplemental Fig. 2), however none were statistically significant after false-discovery rate (FDR) correction. As for higher taxonomic levels, four families and two orders differed between patients and controls, but again none were statistically significant after FDR correction (Supplemental Table 3). When focusing on typical butyric acid producers like the *Lachnospiraceae* and *Ruminococcaceae* families^18^, one genus in the *Ruminococcaceae* family (*Anaerotruncus*) and five genera in the *Lachnospiraceae* family (*Lachnospiraceae UCG_003*, *Eubacterium eligens group*, *Coprococcus*, *Lachnospiraceae CAG_56* and *Ruminococcus gnavus group*) had nominally significant different abundance in patients and controls, but as the first four genera were more abundant in controls, and the latter two in patients, we could not show a consistent pattern (Supplemental Fig. 2). When considering the inferred functional potential to produce butyric acid (as predicted by PICRUSt2), the activity in the dominating pathway for production of butyric acid (pyruvate fermentation to butyric acid)^12^ was increased in the patient group compared with controls (*p* = 0.031).

### Markers of gut barrier damage and inflammasome activation

**Figure 2:**
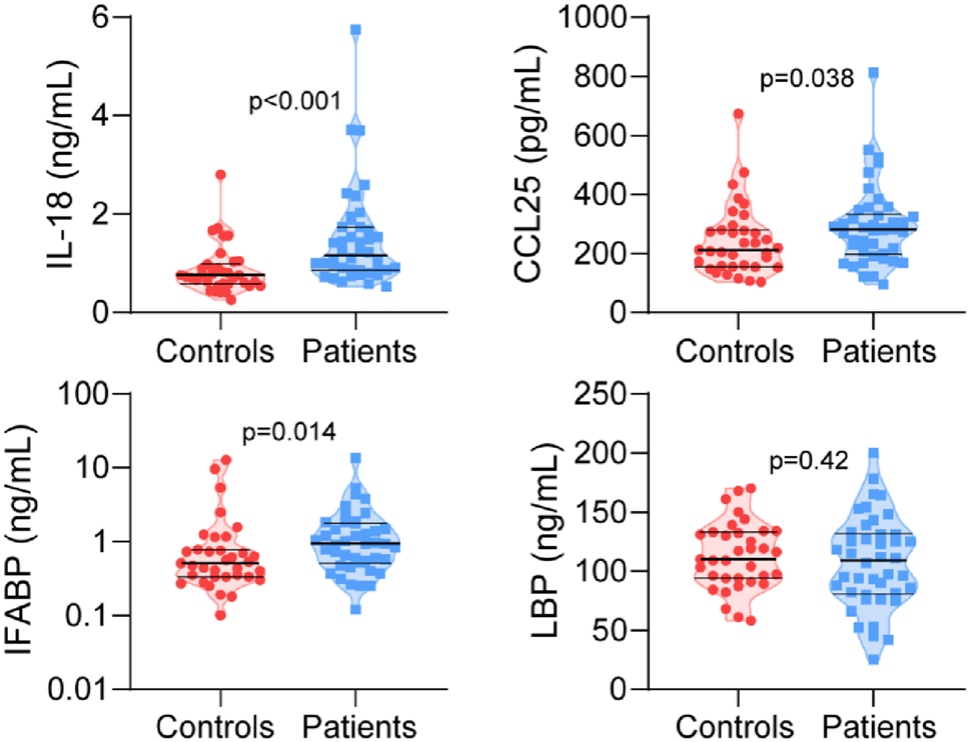
Violin plots of plasma markers of gut barrier damage and inflammasome activation in patients and controls.

Next, markers of gut barrier function and inflammasome activation were assessed (Fig. 2). Level of intestinal fatty acid binding protein (IFABP), reflecting enterocyte damage, was increased in patients compared with controls (0.94 mg/mL (0.12–13.5) vs 0.52 mg/mL (0.10–12.7); *p* = 0.014), while the concentration of lipopolysaccharide binding protein (LBP), reflecting gut leakage, was similar. The gut homing marker chemokine ligand 25 (CCL25) and the marker of inflammasome activation interleukin-18 (IL-18) were also both increased in the patients compared with controls (280 pg/mL (94–813) vs 210 pg/mL (103–673); *p* = 0.038 for CCL25 and 1.2 mg/mL (0.52–5.8) vs. 0.76 mg/mL (0.25–2.8); *p* < 0.001) for IL-18, respectively).

### Correlations between butyric acid, risk factors for stroke, inflammatory markers and gut barrier damage markers in patients and controls

Level of fecal butyric acid correlated with traditional cardiovascular risk factors and inflammatory markers that were elevated in patients compared to controls; including BMI (r = 0.39; *p* < 0.001) and waist-hip ratio (r = 0.26; *p* = 0.024), as well as CRP (r = 0.24; *p* = 0.036), leukocyte count (r = 0.26; *p* = 0.023), and HbA1c (r = 0.30; *p* = 0.016). We found no correlation to hypertension, and no association to dietary intake of fibers or starch.

Increased fecal level was associated with higher abundance of the well-known butyric acid producers *Roseburia* (r = 0.27; *p* = 0.032) and *Faecalibacterium* (r = 0.26; *p* = 0.036), but not correlated to the six genera that differed between our patients and controls. There were no correlations between fecal butyric acid and markers of gut barrier function or inflammasome activation, drugs or dietary intake of fibers or starch.

The dominating pathway for butyric acid production was positively correlated to plasma level of butyric and isobutyric acids (r = 0.38, *p* = 0.020), but not associated to fecal level, nor to dietary intake of fibers or starch.

We found higher levels of IL-18 and CCL25 to be correlated to increased leukocyte count (r = 0.29; *p* = 0.014 and r = 0.28; *p* = 0.018, respectively). IL-18 was positively correlated to IFABP and CCL 25 (r = 0.28; *p* = 0.014 and r = 0.35; *p* = 0.002, respectively).

## Discussion

In this study investigating the association of the SCFA butyric acid to carotid atherosclerosis and other risk factors for ischemic stroke, our main findings were (i) patients with severe carotid atherosclerosis had higher fecal, but not plasma, level of butyric acid compared with healthy controls, (ii) patients had increased activity in the main microbiota-driven pathway for butyric acid production, and (iii) patients had increased plasma levels of markers of gut barrier damage and inflammasome activation compared with controls.

When planning this study we expected to find decreased levels of SCFAs in fecal samples from patients as compared with controls. This would be in keeping with previous evidence on the role of butyric acid exerting local anti-inflammatory effects in the intestinal mucosa^20^ and that loss of butyric acid producing bacteria may result in dysfunctional mucosal barrier, facilitating passive leakage of microbial toxins triggering inflammation and thereby acceleration of atherosclerosis^20–23^. Most previous studies report measured abundance of butyric acid producing bacteria^2–4,24–27^, in patients with atherosclerosis and stroke. To the best of our knowledge, actual levels of fecal SCFAs have not previously been assessed in this population. Somewhat surprisingly, we discovered consistently higher level of butyric acid in patients compared with controls. Similar observations, however, are previously reported by de la Cueste-Zuluaga^28^ studying 440 adults, where higher fecal SCFA concentrations were associated with higher gut permeability, markers of metabolic dysregulation, obesity and hypertension. Schwiertz^29^ and Calderon-Perez^13^ measured fecal SCFAs in obese and hypertensive patients, respectively, and also reported higher levels in patients compared to controls. There are several possible explanations for this finding as diet, microbial production capacity and intestinal absorption, as well as other host factors, are thought to affect fecal levels of SCFA. In our population, we did not identify any significant difference in dietary intake of starch and fibers between the two groups, and it is less likely that diet have influenced our results. Several bacteria have the potential to produce butyric acid. A study by Trefflich comparing fecal SCFAs in 72 vegans and omnivores, nicely showed equal concentrations of SCFAs, but different bacterial clusters to predict concentrations for the two groups^30^. When assessing microbial fermentation capacity for SCFAs in our population, we found no differences in the microbiota composition between the groups. Previously published evidence on the potential for butyric acid production in the gut in CVD is conflicting; most reporting decreased abundance of potential butyric acid producers. Jie^2^ and Zhu^31^ studied a total of 288 patients with coronary artery disease and 285 controls, whereas Karlson^3^ studied 12 patients with symptomatic carotid atherosclerosis and 13 controls, and all showed decreased levels of bacteria identified as butyric acid producers. Tan^24^, Haak^25^ and Li^26^ found reduced level of SCFA producing bacteria in studies comprising in total 568 stroke patients and 241 controls, while Li^27^ found higher abundance of SCFA producers in 30 patients compared with 30 controls. The numerous factors involved in shaping an individual’s microbiota in the short and long time such as diet, medications, co-morbidities, genetics, exposures and timing of the sampling, as well as our small sample size, could influence the lack of differences between our groups. For example, our patient group more frequently used statins and antibiotics, both known to alter microbiota composition. Notably, our understanding of the bacteria with potential to produce SCFAs is also very limited. However, we did find increased activity in the dominating pathway for production of butyric acid (pyruvate fermentation to butyric acid) in patients, suggesting increased production as a cause for higher level of butyric acid in patients.

Looking further into intestinal absorption, we measured markers of gut barrier damage and SCFA levels in plasma. We found significantly increased levels of CCL25 and IFABP, as well as IL-18 in our patient group. Increased intestinal permeability has been reported in multiple human and animal studies of coronary artery disease and atherosclerosis^15^, as well as in ischemic stroke^25^, and a role for gut barrier dysfunction in early stages of disease is evident^2^. Decreased absorption of butyric acid has been proposed as a mechanism of increased fecal level in hypertensive patients^32,33^, and some evidence suggests that increasing intestinal permeability begin before dysbiosis and hypertension^29^. IL-18 was positively correlated to CCL25 and IFABP, suggesting that gut impairment can trigger inflammation, as also described in previous reports^34^.

When assessing the level of butyric and isobutyric acids in plasma, we did not find any significant differences between the two groups. This lack of correlation between SCFA concentrations in feces and plasma is, however, in keeping with other published reports^13,33^. Quantification of SCFAs in plasma is challenging due to their low concentrations^35^, especially for butyric acid being a primary energy source for colonocytes, and this finding must be interpreted with caution. Interestingly, increased activity in the dominating butyric acid production pathway was correlated to higher plasma level of butyric and isobutyric acids, and it is tempting to speculate that this could indicate a compensatory mechanism to increase plasma level, and to repair the gut membrane.

In regards to SCFA levels and traditional risk factors, we found that increased BMI, waist-hip ratio, leukocyte count, CRP and HbA1c were associated with increased level of fecal butyric acid. This is in keeping with a study by Schwiertz^29^ of 68 overweight patients compared to 30 lean controls. Calderon-Perez^13^ showed higher fecal levels of most SCFAs, along with lower plasma levels, in 29 hypertensive patients compared to 32 controls, whereas Verharr^36^ found higher fecal SCFA levels associated with hypertension in a subgroup. However, we could not find correlations to hypertension in our population.

Thus, while the majority of studies show reduced relative abundance of butyric acid producing bacteria across the metabolic syndrome-atherosclerosis disease spectrum^2–4,24–27^, suggesting alleviating effect of butyric acid on high fat diet–induced obesity and insulin resistance^37,38^, the data are not uniform. Studies measuring fecal SCFAs^4,13,29,36^ in related diseases generally report increased levels in patients with CVD. Nevertheless, the role of butyric acid in atherosclerosis is complex and not completely understood, and further studies are needed to unveil the potential role of butyric acid in monitoring disease activity.

This study has several limitations. The study sample is small, fecal samples are collected at different time points in regards to symptoms, hospital admission and carotid endarterectomy.

The patients used significantly more antibiotics, platelet inhibitors and statins than the controls. On the other hand, all blood samples were drawn in the fasting state, all participants were investigated with carotid ultrasound, and we had detailed information on general health measures, diet and anthropometrics. In addition, when accounting for potential confounders known to influence SCFA producers, such as gender^39^ and statins^40^ as well as antibiotics and platelet inhibitors as well as correlating for age, the results remain comparable, although our population was too small too fully evaluate potential interference of gender or the mentioned drugs.

## Conclusion

In contrast to bulk of previous studies showing reduced abundance of butyric acid producing bacteria in patients with atherosclerosis and CVD, we find that patients with severe carotid atherosclerosis and evidence of gut barrier damage have increased fecal level of butyric acid. This finding was supported by increased functional bacterial production of butyric acid. We speculate that gut barrier damage could decrease intestinal absorption, and also influence the results. Further studies are needed to map out the role of butyric acid and other SCFAs in atherosclerosis and their potential future role when considering the gut as a therapeutic target to prevent symptomatic CVD.

## Methods

### Patients and control subjects

**Figure 3:**
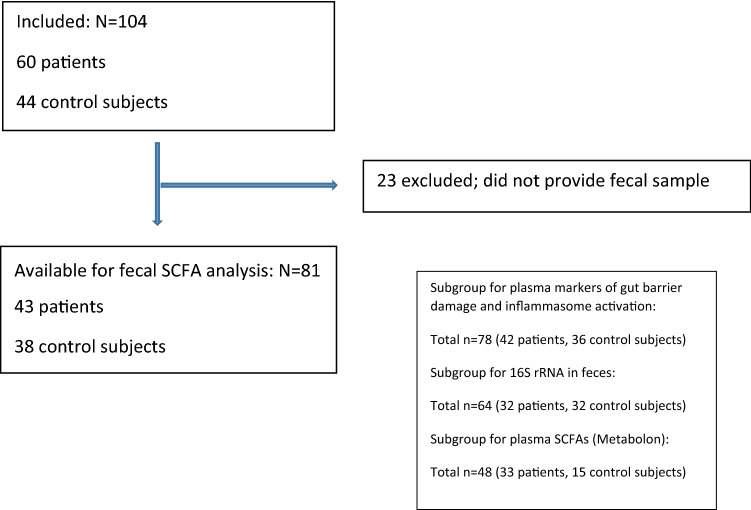
Flow-chart showing inclusion process for participants available for SCFA analysis in feces. Box in lower right corner showing participants available for additional analysis (i.e. plasma markers of gut barrier damage and inflammation activation, 16S rRNA in feces and plasma SCFAs).

Between August 2017 and June 2019, 60 adult patients with severe atherosclerosis; defined as moderate (50–69%) or severe (≥ 70%) carotid stenosis; were consecutively recruited at Oslo University Hospital. Patients with known immunodeficiency or cancer were excluded. For comparison, 44 healthy control subjects were recruited from the same area of Norway as the patients, 12 of these were the patients’ spouses. The controls were healthy individuals with normal findings on carotid ultrasound as well as CRP < 5. A total of 81 participants provided fresh frozen fecal samples available for analysis; 43 patients and 38 control subjects, respectively (Fig. 3).

The study was approved by the Norwegian Regional Committees for Medical and Health Research Ethics (ID REC 2017/2202 A) and was performed in accordance with the Declaration of Helsinki. All the participants gave written informed consent before inclusion.

Our study was registered in ClinicalTrials.gov (NCT04803838). We aimed to show that patients with severe carotid atherosclerosis would have lower level of fecal butyric acid and less butyric acid producing bacteria, evidence of gut dysbiosis and increased inflammation compared to control subjects.

### Carotid ultrasound

Color duplex ultrasound was performed on all participants with a Philips Epiq 5 (Philips, USA), using a Linear probe (3–12 MHz) on both carotid arteries. The degree of internal carotid artery stenosis was determined according to consensus criteria^41^.

### General health and diet

Information regarding previous medical history, risk factors for stroke and medications was collected from a questionnaire and/or medical journals. A validated dietary questionnaire^42^ was completed, waist- and hip circumferences were measured, and weight and height measures submitted by the participants.

### Blood sampling protocol and analysis

Venipuncture of a forearm vein was performed in fasting participants. Blood was drawn into pyrogen-free tubes without any additives and allowed to clot at room temperature (within 1 h) before centrifugation (2500*g* for 20 min). For the gut barrier damage markers, 4 ml Vacutettes with EDTA as anticoagulant were used. All blood samples were stored at − 80 °C until further analysis. Analysis for leukocytes, thrombocytes, total cholesterol HDL, LDL triglycerides, creatinine, GFR, HbA1c and CRP were performed.

### Analysis of plasma SCFAs

Metabolomic profiling was perfomed by Metabolon, Inc. (Durham, NC, USA) using ultrahigh performance liquid chromatography-tandem mass spectrometry (UPLC-MS/MS), as described by Evans^43^. Forty-eight samples, from respectively 33 patients and 15 control subjects were analyzed on Metabolon’s global HD4 platform and the complex lipids panel (CLP). Lipids were extracted from the biofluid in the presence of deuterated internal standards using an automated BUME extraction^44^. The lipids and fatty acids were detected as described by the provider using ammonium acetate dichloromethane:methanol (50:50), followed by infusion-MS analysis, performed on a Shimazdu LC with nano PEEK tubing and the Sciex SelexIon-5500 QTRAP. Individual lipid species were quantified by taking the peak area ratios of target compounds and their assigned internal standards, then multiplying by the concentration of internal standard added to the sample^45^. Lipid species concentrations were background-subtracted using the concentrations detected in process blanks (water extracts). The resulting background-subtracted, run-day normalized lipid species concentrations were then used to calculate the lipid class and fatty acid total concentrations, as well as the mol% composition values for lipid species, lipid classes, and fatty acids.

### Analysis of markers of gut barrier damage and inflammasome activation

Plasma levels of IFABP, CCL25, IL-18, and LBP were measured in duplicate by enzyme immunoassays (EIA) using commercially available antibodies (R&D Systems, Minneapolis, MN, USA) in a 384 format using a combination of a SELMA (Jena, Germany) pipetting robot and a BioTek (Winooski, VT, USA) dispenser/washer. Absorption was read at 450 nm with wavelength correction set to 540 nm using an EIA plate reader (Bio-Rad, Hercules, CA, USA). All samples for a marker were run on the same 384 plate and intrassay coefficient of variation was < 10%.

### Fecal sample collection and storage

Study participants sampled feces at home by defecating into a clean device and then transferring into provided clean tubes without additives. Samples were frozen immediately at − 20 °C, brought to the hospital in a cooling device, and subsequently frozen at − 80 °C. Participants were instructed to freeze their samples immediately. Storage time at − 20 °C ranged between 1 and 14 days for both patients and controls. Some patients submitted samples during the hospital stay, and samples were stored at a maximum of 4 h in a standard refrigerator before frozen at − 80 °C. The participants evaluated their stools according to the Bristol stool scale^46^.

### Analysis of fecal SCFAs

Fecal samples (0.5 g) and distilled water containing 3 mmol/L of 2-ethylbutyric acid (as internal standard) and 0.5 mmol/L of H_2_SO_4_ were homogenized. 2.5 mL of the homogenate was vacuum distilled according to the method of Zijlstra et al.^47^ and modified by Høverstad et al.^48^. The distillate was analyzed with gas chromatography (Agilent 6850; Agilent, CA, USA) using a capillary column (serial no. USE400311H, Agilent J&W GC columns; Agilent, CA, USA) and quantified while using internal standardization. Flame ionization detection was employed. The total amount of all SCFAs and the amount of acetic, propionic, butyric, isobutyric, valeric, isovaleric, caproic, and isocaproic acids expressed in mmol/Kg wet weight were measured.

### Analysis of gut microbiota composition and function

Fecal DNA was extracted using the commercial ZymoBIOMICS™ DNA Miniprep Kit (ZR, Zymo Research, Irvine, CA, USA), according to manufacturer's instructions, with slight modifications.

Libraries for 16S rRNA amplicon sequencing were prepared as previously described^49^. Briefly, the hypervariable regions V3 and V4 of the 16S rRNA gene were amplified using dual-indexed universal primers (319F:ACTCCTACGGGAGGCAGCAG and 806R:GGACTACHVGGGTWTCTAAT) and Phusion High-Fidelity PCR Master mix m/HF buffer (Thermo Fisher Scientific, USA). The PCR products were cleaned and normalized using the SequalPrep Normalization Plate Kit (Thermo Fisher Scientific, USA). Quality control and quantification of the pooled libraries were performed using an Agilent Bioanalyzer (Agilent Technologies, USA) and Kapa Library Quantification Kit (Kapa Biosystems, London, UK). Sequencing was performed at the Norwegian Sequencing Centre (Oslo, Norway), using the Illumina MiSeq platform and v3 kit (Illumina, San Diego, CA, USA), set at 300 base pair paired-end reads.

Paired-end reads were filtered for Illumina Universal Adapters and PhiX, demultiplexed, quality trimmed and merged using BBDuk 38.90^50^, Cutadapt 3.3^51^ and BBMerge 38.90^52^. Denoising reads to Amplicon Sequence Variants (ASVs), taxonomic classification, filtering of contaminants and rare ASVs and building of a phylogenetic tree was done with QIIME2 version 2021.2^53^. To reduce the effect of uneven sequencing depths, we rarefied all samples to a common level of 10,600 counts. We calculated diversity metrics in QIIME2 and tested for differential abundance of genera using this rarefied dataset (Mann–Whitney U test). Before differential abundance testing we applied a taxa prevalence filter of 20% on the rarefied dataset. To assess the functional characteristics of the gut microbiota, we performed an analysis with PICRUSt2^54^ in QIIME2 to predict the metagenomic content in each sample and tested for differentially abundant pathways with aldex2 (with prevalence filter 20%).

### Statistics

Descriptive statistics are given as number and proportion (%), mean with standard deviations or median (min–max). Mann–Whitney U test was used to compare the non-parametric categorical variables with continuous variables. The Spearman’s rank correlation test was used to evaluate relationships between variables.

*P*-values are two-sided and considered significant when < 0.05. IBM SPSS Statistics for Windows, statistical software version 25.0 (IBM Corp., Armonk, NY, USA) was used for data analyses.

## Supplementary Information

Supplementary Information.

## Data Availability

The data from the medical and dietary questionnaire is available upon request to corresponding author Kristine Stø. Due to data protection regulations and lack of consent, the data are not deposited in public repositories. However, data will be available upon request to the corresponding author, pending a material and data transfer agreement and an amendment to the Regional committee for medical and health research ethics.
