# Supplementary material for: Fecal level of butyric acid, a microbiome-derived metabolite, is increased in patients with severe carotid atherosclerosis
Source: Sci Rep. 2022 Dec 26;12:22378. doi: 10.1038/s41598-022-26759-x (PMC9792531; doi:10.1038/s41598-022-26759-x)

Supplementary material

Title: Fecal level of butyric acid, a microbiome-derived metabolite, is increased in patients with severe carotid atherosclerosis

Authors: Kristine Stø[1,2], Jørgen Valeur[3], Thor Ueland[1,4], Gunn Helen Malmstrøm[3], Vigdis Bjerkeli [1], Marius Trøseid[1,4,5], Johannes R. Hov[1,4,6,7], Kristian Holm[1,4,7], Beate Vestad[4,7], Bente Halvorsen[1,4], Mona Skjelland[1,2], Karolina R. Skagen[2].

Supplemental table 1: Baseline characteristics in subgroups of patients with 16S rRNA analysis and plasma SCFA analysis.

Supplemental table 2: Table comparing levels of butyric acid and relevant butyric acid producing bacteria (genus level) between sexes and users and nonusers of statins, antibiotics and platelet inhibitors, as well as correlation with age. Analysis were restricted to patients so that the groups would be comparable. The only significant difference we observed was for *Lachnospiraceae UCG_003* being higher in users of antibiotics.

Supplemental table 3: Table showing differential abundance for bacterial genera (a), families (b) and orders (c) that differed between patients and controls, before and after FDR correction.

Supplemental figure 1: Barplot showing top 10 bacterial genera (average abundance) in patients and controls.

Supplemental figure 2: Barplots of bacteria (genus-level) that differed between patients and controls.

Supplemental table 1: Baseline characteristics in subgroups of patients with 16S rRNA analysis and plasma SCFA analysis.

	16S rRNA population			Metabolon (plasma SCFA) population		
	Patients N=32	Controls N=32	*p*-value	Patients N=33	Controls N=15	*p*-value
Age, (years) *	73 (7)	67 (8)	0.002	72 (7)	71 (3)	0.224
Male sex	44 (14)	25 (8)	0.114	45 (15)	47 (7)	0.938
Body Mass Index (kg/m^2)	24.9 (3.7)	24.6 (3.0)	0.816	26.2 (4.0)	24.4 (3.4)	0.129
Waist-hip ratio (cm/cm)	0.95 (0.09)	0.89 (0.07)	0.012	0.97 (0.08)	0.91 (0.06)	0.018
Hypertension	87.5 (28)	21.9 (7)	<0.001	84.8 (28)	20.0 (3)	<0.001
Type 2 Diabetes mellitus	25.0 (8)	0 (0)	0.002	27.3 (9)	0 (0)	0.025
Hypercholesterolemia	68.8 (22)	18.8 (6)	<0.001	57.6 (19)	6.7 (1)	0.001
Anti-platelet treatment	78.1 (25)	9.4 (3)	<0.001	81.8 (27)	6.7 (1)	<0.001
Statin treatment	81.3 (26)	9.4 (3)	<0.001	78.8 (26)	0 (0)	<0.001
C-reactive protein, (mg/L)*	3.3 (5.6)	1.3 (1.0)	0.256	3.7 (5.7)	1.3 (1.1)	0.124
Leukocyte count, (10^9/L)*	7.9 (2.0)	5.0 (1.1)	<0.001	8.0 (2.0)	5.1 (1.2)	<0.001
Total cholesterol, (mM)*	4.1 (1.0)	5.3 (0.88)	<0.001	3.9 (1.0)	5.6 (1.0)	<0.001
LDL cholesterol, (mM)*	2.2 (0.9)	3.2 (0.8)	<0.001	2.1 (0.8)	3.4 (1.0)	<0.001
HbA1c (%)*	5.8 (0.9)	5.2 (0.3)	0.010	5.9 (1.0)	5.3 (0.3)	0.012
Antibiotics last 3 months	25.0 (8)	0 (0)	0.002	27.3 (9)	0 (0)	0.025

Values are given as % (n) or *mean (SD).

Supplemental table 2: Table comparing levels of butyric acid and relevant butyric acid producing bacteria (genus level) between sexes and users and nonusers of statins, antibiotics and platelet inhibitors, as well as correlation with age. Analysis were restricted to patients so that the groups would be comparable. The only significant difference we observed was for *Lachnospiraceae UCG_003* being higher in users of antibiotics.

	Sex			Statins			Antibiotics last 3 months			Platelet inhibitor			Correlation with age*
	women	men	p	yes	no	p	yes	no	p	yes	no	p	
Faecalibacterium	7,7E-02	7,2E-02	0,676	7,4E-02	7,8E-02	0,866	7,3E-02	7,6E-02	0,931	7,5E-02	7,6E-02	0,802	0.02
Roseburia	1,5E-02	1,2E-02	0,704	1,5E-02	1,2E-02	0,923	1,1E-02	1,5E-02	0,528	1,2E-02	2,2E-02	0,101	-0.05
anaerotruncus	2,3E-04	1,6E-04	0,617	2,3E-04	6,3E-05	0,178	2,4E-04	1,9E-04	0,114	2,3E-04	9,4E-05	0,457	0.17
ruminococcus_gnavus	5,3E-01	6,7E-01	0,063	7,9E-03	3,1E+00	0,648	2,0E-03	7,9E-01	0,355	7,6E-01	0,0E+00	0,038	-0.32
coprococcus	6,4E-03	8,7E-03	0,371	6,4E-03	1,2E-02	0,184	6,1E-03	7,8E-03	0,472	7,2E-03	7,9E-03	0,273	-0.25
eubacterium_eligens	2,3E-03	2,0E-03	0,818	1,8E-03	3,7E-03	0,981	1,3E-03	2,5E-03	0,114	1,7E-03	4,0E-03	0,27	0.26
CAG_56	1,5E-03	1,5E-03	0,898	1,6E-03	1,1E-03	0,143	9,2E-04	1,7E-03	0,353	1,3E-03	2,3E-03	0,343	0.32
Lachnospiraceae UCG_003	1,5E-04	6,7E-05	0,892	3,6E-05	4,6E-04	0,227	**4,6E-04**	**0,0E+00**	**0,013**	1,5E-04	0,0E+00	0,447	0.06
Fecal butyric acid	13,29	9,78	0,056	11,62	12,17	0,834	12,28	11,59	0,571	12,61	7,93	0,142	-0.14

*Spearman correlation, no significant findings

Supplemental table 3: Table showing differential abundance for bacterial genera (a), families (b) and orders (c) that differed between patients and controls, before and after FDR correction.

a) Mann-Whitney on rarefied data (genus level) Wilcoxon (rarefied): control_patient, 1 vs 2

Genus	mean_1	mean_2	Wilcoxon_pvalue	Wilcoxon_pvalue_fdr
Lachnospirales;f__Lachnospiraceae;g__Frisingicoccus	0.0000767	0.0006014	0.0021023	0.1881198
Lachnospirales;f__Lachnospiraceae;g__Lachnospiraceae_UCG-003	0.0010937	0.0001150	0.0028881	0.1881198
Lachnospirales;f__Lachnospiraceae;g__[Eubacterium]_eligens_group	0.0044870	0.0021846	0.0040025	0.1881198
Lachnospirales;f__Lachnospiraceae;g__Coprococcus	0.0128626	0.0073880	0.0105049	0.3702969
Gastranaerophilales;f__Gastranaerophilales;g__Gastranaerophilales	0.0043986	0.0004127	0.0238752	0.3870909
Erysipelotrichales;f__Erysipelotrichaceae;g__[Clostridium]_innocuum_group	0.0000472	0.0002889	0.0242896	0.3870909
Desulfovibrionales;f__Desulfovibrionaceae;g__uncultured	0.0002506	0.0001032	0.0306311	0.3870909
Peptostreptococcales-Tissierellales;f__Peptostreptococcaceae;g__Romboutsia	0.0085171	0.0026209	0.0325404	0.3870909
Oscillospirales;f__UCG-010;g__UCG-010	0.0098143	0.0040360	0.0330891	0.3870909
Lachnospirales;f__Lachnospiraceae;g__CAG-56	0.0018779	0.0014947	0.0339882	0.3870909
Victivallales;f__Victivallaceae;g__Victivallis	0.0002889	0.0000796	0.0343061	0.3870909
Oscillospirales;f__Ruminococcaceae;g__Anaerotruncus	0.0001032	0.0001975	0.0346723	0.3870909
Lachnospirales;f__Lachnospiraceae;g__[Ruminococcus]_gnavus_group	0.0007488	0.0066156	0.0356892	0.3870909

b) Mann-Whitney on rarefied data (Family level) Wilcoxon (rarefied): control_patient, 1 vs 2

Family	mean_1	mean_2	Wilcoxon_pvalue	Wilcoxon_pvalue_fdr
o__Peptostreptococcales-Tissierellales;f__Peptostreptococcaceae	0.0137588	0.0055896	0.0201475	0.421886
o__Gastranaerophilales;f__Gastranaerophilales	0.0043986	0.0004127	0.0238752	0.421886
o__Victivallales;f__Victivallaceae	0.0003361	0.0000884	0.0328090	0.421886
o__Oscillospirales;f__UCG-010	0.0098143	0.0040360	0.0330891	0.421886

c) Mann-Whitney on rarefied data (Order level) Wilcoxon (rarefied): control_patient, 1 vs 2

Order	mean_1	mean_2	Wilcoxon_pvalue	Wilcoxon_pvalue_fdr
o__Peptostreptococcales-Tissierellales	0.0183166	0.0093072	0.0113657	0.3296044
o__Gastranaerophilales	0.0043986	0.0004127	0.0238752	0.3461906

Supplemental figure 1: Barplot showing top 10 bacterial genera (average abundance) in patients and controls.

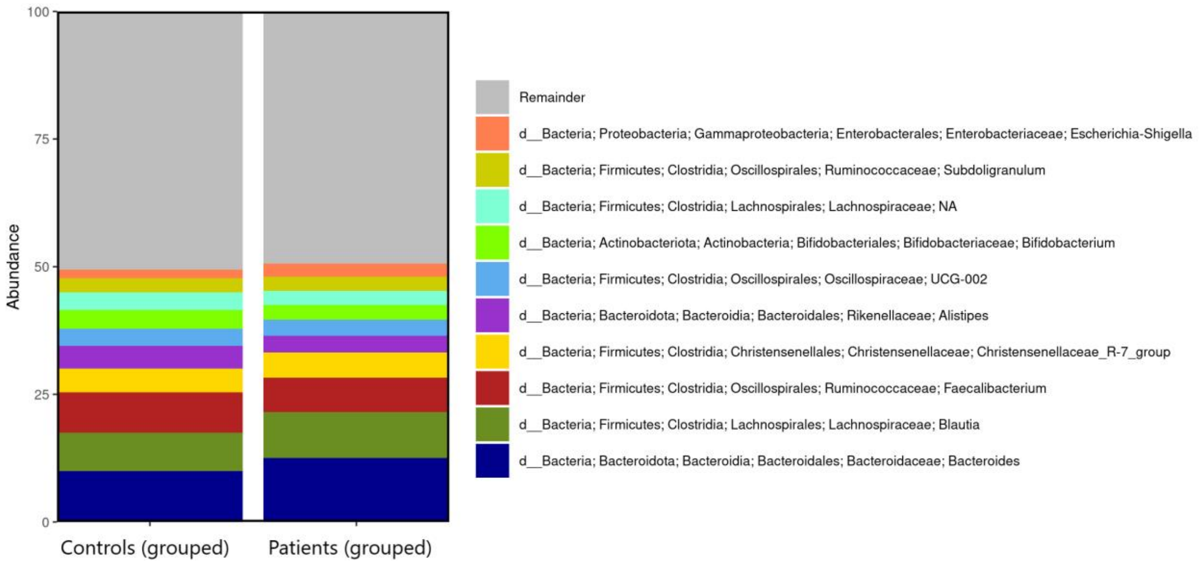

Supplemental figure 2: Barplots of bacteria (genus-level) that differed between patients and controls.

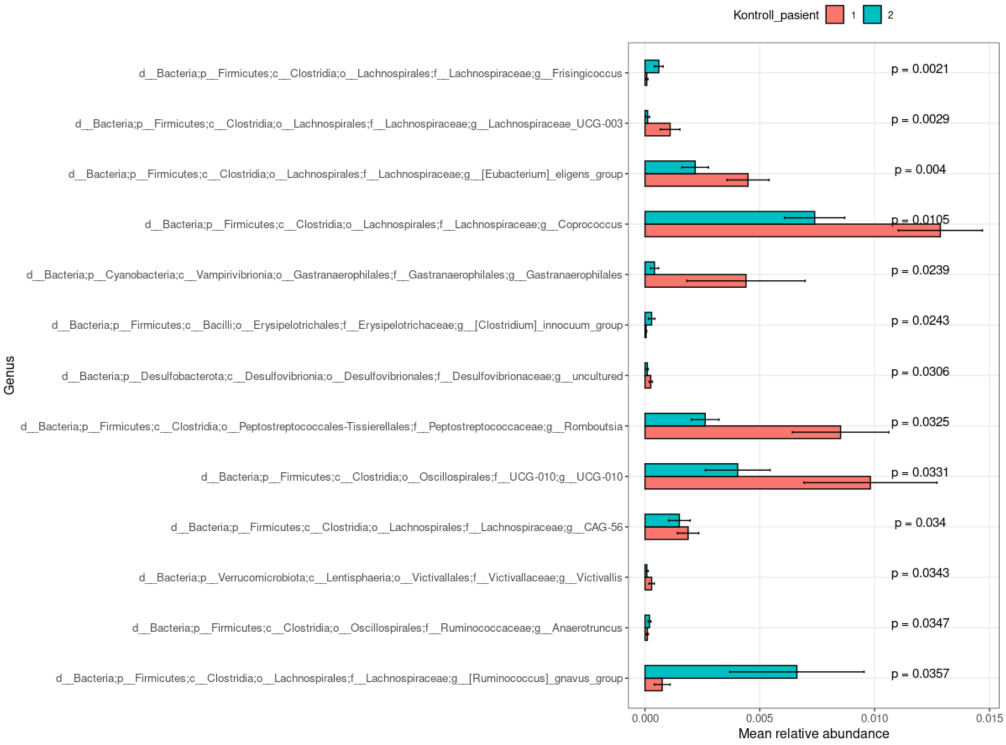

Supplement: Supplementary file 1 — Supplementary Information. [file 41598_2022_26759_MOESM1_ESM.pdf]
